# Omega-3 and Postpartum Depression: Assessing the Effectiveness of Omega-3 Polyunsaturated Fatty Acids in Preventing Postpartum Depression

**DOI:** 10.1192/j.eurpsy.2025.1363

**Published:** 2025-08-26

**Authors:** F. M. A. Santos, P. Coelho, P. Jorge, A. Silva, R. Lopes, V. Melo, C. Rodrigues, C. Matias, T. Vieira, L. Delgado, F. Costa, V. Santos

**Affiliations:** 1 Department of Psychiatry and Mental Health, ULS Médio Tejo, Tomar; 2 Department of Psychiatry and Mental Health, Cova da Beira Local Health Unit; 3 Faculty of Health Sciences, University of Beira Interior, Covilhã, Portugal

## Abstract

**Introduction:**

Postpartum depression (PPD) affects approximately 9% of new mothers and can have profound consequences on both maternal well-being and infant development. Additionally, around 50% of women experience depressive symptoms or mood disturbances during pregnancy and lactation. A growing body of research suggests a potential link between omega-3 polyunsaturated fatty acids (PUFAs) and mood regulation, particularly in the prevention of PPD. Omega-3 PUFAs, primarily docosahexaenoic acid (DHA) and eicosapentaenoic acid (EPA), are found in high concentrations in oily fish and seafood. Over the past century, dietary intake of omega-3 fatty acids has significantly declined in industrialized societies, coinciding with increased rates of mood disorders. Notably, populations with higher fish consumption, such as those in Hong Kong, China, and Japan, report lower incidences of depression compared to Western countries, where lower fish consumption has been linked to higher rates of PPD. During pregnancy and lactation, women become particularly depleted in omega-3 PUFAs, as these essential fats are preferentially diverted to support fetal development, potentially increasing the risk of depression in mothers.

**Objectives:**

This study aims to explore the effectiveness of omega-3 PUFAs in preventing PPD.

**Methods:**

A comprehensive literature review was conducted to evaluate the effectiveness of Omega-3 in preventing PPD. The search was performed on PubMed using key terms such as “Omega-3,” “Postpartum Depression,” and “Prevention.”

**Results:**

In one study, women with low LCP intake were supplemented with 220 mg of DHA, which had no effect on depression, like in another study that reported no effect of 1,8 g of Omega-3 PUFAs on maternal depressive symptoms. Another trial involving 2,399 women found no significant difference in depressive symptoms between those receiving 800 mg of DHA and a control group during the first six months postpartum. A randomized controlled trial of 126 women at risk PPD also showed no benefit from EPA or DHA supplementation in reducing Beck Depression Inventory (BDI) scores compared to placebo. A meta-analysis of 11 trials (N = 3,181) similarly found no significant difference in depression scores between Omega-3 and placebo groups, and no association between the daily doses of DHA or EPA and PPD prevention

**Conclusions:**

Based on the reviewed trials, there is insufficient evidence to recommend Omega-3 fatty acid supplementation (DHA and EPA) at any dosage for the prevention of postpartum depression.

**Disclosure of Interest:**

None Declared

